# Prostate Cancer—Research Advances in Early Detection

**DOI:** 10.3390/jcm14093067

**Published:** 2025-04-29

**Authors:** Aleksandra Czerw, Andrzej Deptała, Mariola Głowacka, Olga Partyka, Monika Pajewska, Natalia Czerw, Anna Badowska-Kozakiewicz, Katarzyna Sygit, Zygmunt Kopczyński, Piotr Czarnywojtek, Izabela Gąska, Mateusz Kaczmarski, Tomasz Banaś, Elżbieta Grochans, Szymon Grochans, Anna M. Cybulska, Daria Schneider-Matyka, Ewa Bandurska, Weronika Ciećko, Grażyna Dykowska, Zofia Sienkiewicz, Jarosław Drobnik, Piotr Pobrotyn, Urszula Grata-Borkowska, Joanna Furtak-Pobrotyn, Aleksandra Sierocka, Michał Marczak, Dagmara Pokorna-Kalwak, Remigiusz Kozlowski

**Affiliations:** 1 Department of Health Economics and Medical Law, Medical University of Warsaw, 01-445 Warsaw, Poland; 2 Department of Economic and System Analyses, National Institute of Public Health NIH-National Research Institute, 00-791 Warsaw, Poland; 3 Department of Oncology Propaedeutics, Medical University of Warsaw, 01-445 Warsaw, Poland; 4 Institute of Nursing, Nursing Department, Faculty of Health Science, Collegium Medicum, Masovian University in Płock, 09-402 Płock, Poland; 5 Students’ Scientific Organization of Cancer Cell Biology, Department of Oncology Propaedeutics, Medical University of Warsaw, 01-445 Warsaw, Poland; 6 Faculty of Health Sciences, Calisia University, 62-800 Kalisz, Poland; 7 Medical Institute, Jan Grodek State University in Sanok, 38-500 Sanok, Poland; 8 Department of Radiotherapy, Maria Sklodowska-Curie Institute-Oncology Center, 31-115 Cracow, Poland; 9 Department of Nursing, Faculty of Health Sciences, Pomeranian Medical University in Szczecin, 71-210 Szczecin, Poland; 10 Department of Pediatric and Oncological Surgery, Urology and Hand Surgery, Faculty of Medicine and Dentistry, Pomeranian Medical University in Szczecin, 71-252 Szczecin, Poland; 11 Center for Competence Development, Integrated Care and e-Health, Medical University of Gdansk, 80-204 Gdansk, Poland; 12 Institute of Nursing, College of Engineering and Health, 02-366 Warsaw, Poland; 13 Department of Nursing, Social and Medical Development, Medical University of Warsaw, 01-445 Warsaw, Poland; 14 Department of Family Medicine, Faculty of Medicine, Wroclaw Medical University, 51-141 Wroclaw, Poland; 15 Remedial Specialistic Clinic, “Pulsantis Sp z o.o”, 53-238 Wroclaw, Poland; 16 Citodent Dental Center Furtak-Pobrotyn &Company Limited Partnership, 05-220 Olawa, Poland; 17 Department of Management and Logistics in Healthcare, Medical University of Lodz, 90-131 Lodz, Poland; 18 Department of Innovation of Merito University in Poznan, 61-895 Poznan, Poland

**Keywords:** prostate cancer, screening, clinical trial

## Abstract

Screening is widely considered one of the most effective methods for the early detection of prostate cancer. Early detection allows for prompt treatment, which increases the likelihood of a full recovery. This review sought to summarize studies on screening methods related to prostate cancer. It examined trends in screening practices and organized the findings into two main categories. The first category focused on strategies to boost screening participation, reach underserved populations, and improve public awareness of health issues. The second category concentrated on refining current diagnostic methods, developing new tests, and testing biomarkers. A significant portion of the research also explored diagnostic techniques aimed at enhancing patient comfort during exams without compromising clinical effectiveness.

## 1. Introduction

According to information provided by the World Health Organization, prostate cancer is, after lung cancer, the second most common malignant cancer among men. Global incidence in 2022 was 1,467,854 cases [1]. The age-standardized incidence rate was 29.4/100,000. The highest incidence rate was recorded in France at 157.5/100,000. In terms of mortality, prostate cancer ranks fifth in men. In 2022, 397,430 cases were recorded worldwide. The age-standardized mortality rate was 7.3/100,000. The European Commission estimates the risk of developing prostate cancer up to the age of 74 as 1:11 [2]. The probability of five-year survival is high in countries such as Austria, Germany, Finland, Belgium, France and Portugal, around 90%. The probability of five-year survival is much lower, i.e., in the range of 60–70%, in countries such as Denmark, Latvia, Poland and Slovakia. The lowest probability is observed in Bulgaria, i.e., in the range of 50–60%.

Risk factors for prostate cancer include age, black race, a history of prostate cancer in the immediate family (brother, father), BRCA2 mutations and Lynch syndrome [3]. There is no clear association between smoking and prostate cancer, but smoking men diagnosed with prostate cancer have a worse prognosis than non-smoking men [4]. The correlation between alcohol consumption and prostate cancer is not clear and depends on the amount and type of alcohol consumed [5].

Diagnostics and screening tests include a digital rectal examination (DRE), a prostate-specific antigen (PSA) test, and a transrectal ultrasound of the prostate gland, as well as magnetic resonance imaging (MRI).

The U.S. Preventive Services Task Force (USPSTF) recommends individual decisions regarding participation in PSA testing for men aged 55–69 based on the patient’s family history, ethnicity and comorbidities. PSA testing is not recommended for patients aged 70 and older [6]. The European Association of Urology recommends early testing with PSA from the age of 50. However, for men with a family history of PCa and men of African descent, the corresponding age for testing is 45, while for men carrying BRCA2 mutations, it is lowered to 40 [7]. Similarly, the European Society for Medical Oncology recommends early PSA testing for men > 50, men > 45 with a family history of prostate cancer, African Americans > 45 and BRCA1/2 carriers > 40 [8].

Primary prevention of prostate cancer is based on a diet rich in polyunsaturated fatty acids, vegetables and fruits containing selenium, vitamins D, E and beta-carotene, regular physical activity and avoiding a sedentary lifestyle [9].

## 2. Materials and Methods

The objective of this study is to present current trends in clinical trials for prostate cancer screening.

The basis for the analysis was information collected from the largest global registry of clinical trials (ClinicalTrials.Gov). This registry is maintained by the American National Institute of Health and the National Library of Medicine. ClinicalTrial.Gov is the largest database containing information on over 507,000 clinical trials conducted in over 220 countries worldwide [10]. Due to the subject matter covered, only those studies that referred to prostate cancer were included in the analysis.

The analysis included interventional and observational studies with the primary goal of early prostate cancer detection. To present the current trend in studies, the search period was limited to 2019–2024. Studies that were terminated and active by 4 September 2024 were included in this analysis. It should be noted that the analyzed study covered the period of the COVID-19 pandemic, which had an impact on the level of cancer screening participation [11].

**Figure 1:**
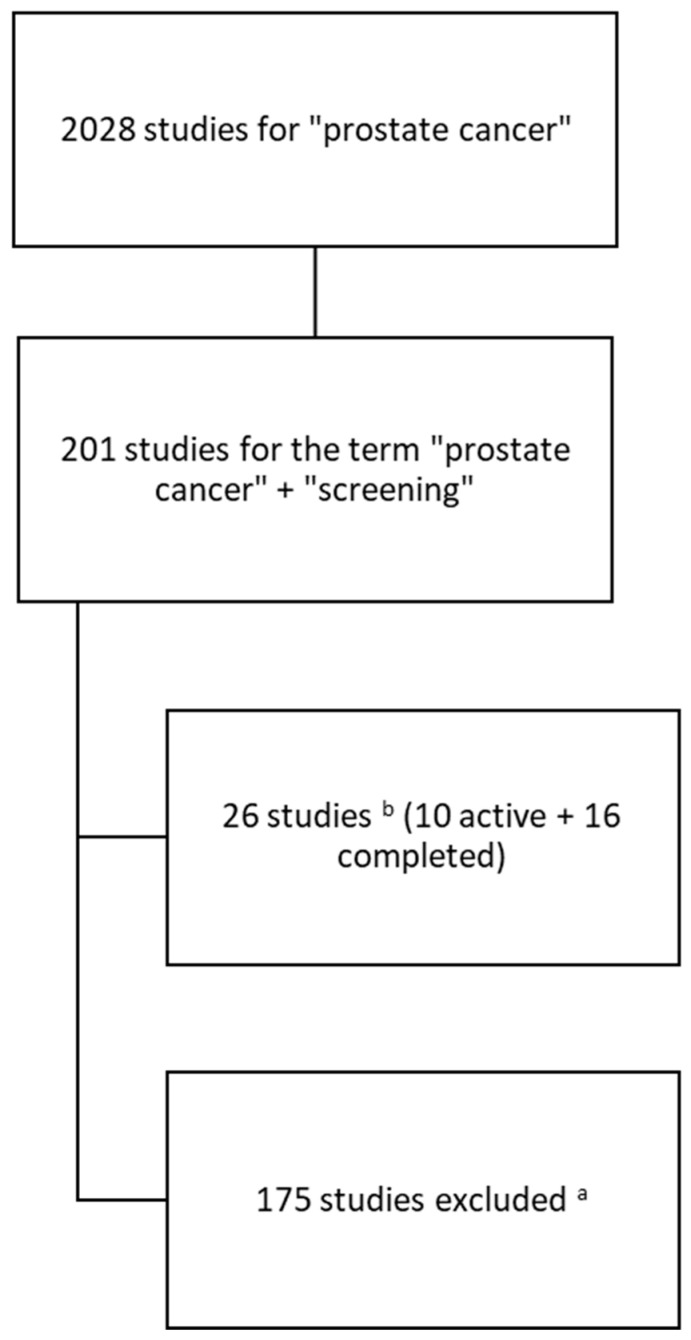
Scheme of search and inclusion of studies in the analysis; ^a^—studies with status: terminated, not yet recruiting, recruiting or other; ^b^—in the period of time of 1 January 2019–4 September 2024.

The process tree for including studies in the analysis is presented below (Figure 1). We started with the key phrase “prostate cancer” and then added the term “screening”. We excluded studies that were terminated, meaning studies that were registered, but not carried out. Also, we excluded studies that were still at a very early stage, i.e., recruiting or not yet recruiting. This allowed us to focus on studies that were either completed or at an advanced stage. As a result, we identified a total of 26 studies, of which 10 were active and 16 were completed.

## 3. Results

Table 1 presents the results of the analysis of the collected materials regarding studies on the early detection of prostate cancer in the period under review.

**Figure 2:**
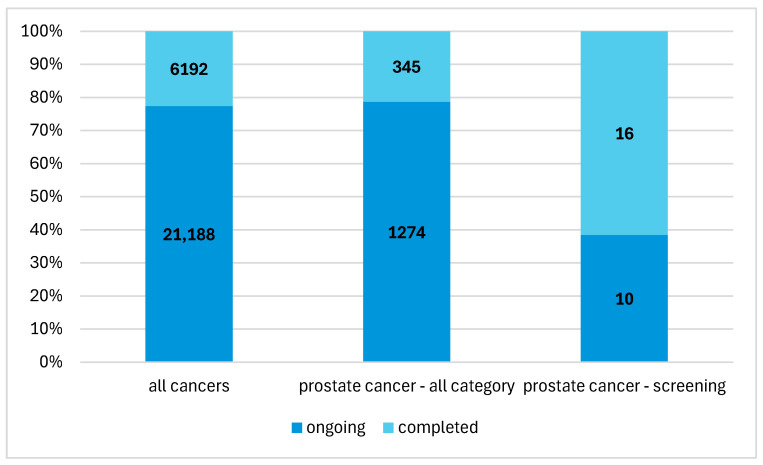
The percentage share of early prostate cancer detection studies in the structure of overall cancer research.

In the entire database, there were 27,380 studies on cancer registered in the years 2019–2024. Of this number, studies on prostate cancer accounted for 5.9% (*n* = 1619) (Figure 2). This group also included studies on the therapeutic management of diagnosed patients, studies on new drugs and studies on patients in remission. Studies on the early detection of prostate cancer accounted for 9.9% (*n* = 201) of all studies on prostate cancer.

Of the twenty-six studies, ten were initiated in 2019, eight in 2020, six in 2021, one in 2022, and one in 2023. Twenty-one studies were interventional studies, and the remaining five studies were observational studies. Twenty-five studies involved adults only. Sample sizes ranged from six to four thousand seven hundred participants. Eleven studies involved behavioral interventions, and fifteen studies involved diagnostic tests.

We start our review with the studies focused on behavioral interventions. In the study conducted since 2021, which is still ongoing (1.), guidelines were developed for men with elevated PSA (4.0 ng/mL) regarding the optimal age of patients who should undergo regular PSA testing, the optimal frequency of repeat testing and the PSA threshold value that should be an indication for biopsy [12]. Another study (2.) showed that patients involved in the decision-making process regarding diagnostic procedures mostly decide to participate in PSA testing [13]. This study has already been completed. The effectiveness of engaging adult African American men in the decision-making process regarding their diagnostics, conducted within a community treatment setting, was also demonstrated in another completed study (3.) [14]. Men participating in the program had greater knowledge about prostate cancer and were willing to make decisions about their participation in testing based on this knowledge. An evaluation of the effectiveness of multimedia education was also conducted in relation to the population of African American men (4.) [15]. Patients have been shown to trust prostate cancer information presented in online videos provided by physicians more than by other patients (5.) [16]. Studies conducted since 2023 (6.), still ongoing, are verifying the effectiveness of an app that provides information on risk factors, diagnostic procedures and symptoms of prostate cancer, also targeted at the African American male population. The app also provides access to a PSA test that can be performed at home [17]. Studies conducted since 2020 (7.), still active, are also analyzing the effectiveness of an educational program for African American men, in which information about prostate cancer is provided by people who are not medical personnel but are peers of people participating in tests [18]. Two studies also analyzed the treatment of patients living in areas with limited access to medical services in the area of cancer diagnostics, including prostate cancer, conducted as part of community treatment (8.) and primary care (9.) [19,20].

One of the risk factors for cancer is the occurrence of such diseases in the patient’s family. A study is being conducted (10.), which aims to verify the effectiveness of an app used by both patients and physicians, which allows the collection of medical data regarding patients’ families and makes it available to the physician before the appointment [21]. This study is still active.

Evaluation in the studies currently being conducted (11.) also includes internet-based intervention regarding the diet and physical activity of patients with already diagnosed cancer, including prostate cancer. The effectiveness of the intervention will be analyzed in the context of quality of life, physical fitness and use of medical services [22].

Studies focused on diagnostic tests are another group of studies. The studies conducted in 2019–2023 (12.) concerned the evaluation of the organizational effort and costs of conducting screening tests, which would be based on an MRI examination [23]. The subject of the study conducted in 2019–2021 (13.) was also to assess the effectiveness of the diagnostic procedure, in which internists referred patients for MRIs. The results were then used to diagnose prostate cancer [24]. Another study (14.) demonstrated a higher diagnostic value of biopsy (so-called fusion biopsy) performed using MRI compared to biopsy (so-called sampling) performed using a transrectal ultrasound [25]. The results of both methods were assessed using a comparison to the results of the histopathology. When looking at cells under a microscope, grades between 1 and 5 were assigned, where 1 meant normal and 5 meant very different from normal. Adding the two most common grades determined the Gleason score. Higher numbers indicated a faster growing cancer. When comparing two groups, the number of participants with a Gleason score equal to 7 or higher was equal to 40.7% in the MRI group and to 38.1% in the ultrasound group. The number of biopsy samples with a Gleason score equal to 7 or higher was equal to 5.2% in the MRI group and to 1.2% in the ultrasound group. When using a structured category assessment system for prostate mpMRI called PI-RADS 2.0, with categories 4 (high) or 5 (very low) for a clinically significant PCa to be present, the accuracy of the mpMRI in correctly characterizing a Gleason 7 or higher prostate cancer, according to histopathology, was equal to 85.2%, while the accuracy in the ultrasound group was equal to 23.8%.

A still-active study (15.) is being conducted to develop a genetic profile (using the SNP technique) of men at an increased risk of prostate cancer. One hundred and seventy polymorphisms are being analyzed in conjunction with MRI and biopsy results to develop a genetic profile of increased risk for use in screening diagnostics [26].

The use of 3D MRI in prostate cancer diagnostics is also being evaluated compared to the standard MRI in another ongoing study (16.) [27]. Another study (17.) has investigated the possibility of combining PET and CT scans for a more accurate diagnosis of metastases in unresectable prostate cancer [28]. The sensitivity of a transperineal prostate biopsy guided by MRI scanning compared to a transrectal prostate biopsy guided by MRI scanning is also being analyzed (18.). A transperineal biopsy performed under local anesthesia offers hope not only for greater sensitivity in detecting cancer but also for reducing the number of infections [29].

The diagnostic value of the test based on a telomere analysis is being analyzed in comparison with the diagnostic value of the PSA test (19.). Initial results have shown that the test based on a telomere analysis is characterized by greater specificity and allows for a reduction in the number of biopsies performed [30].

Another study (20.) showed greater sensitivity of PET using the radiopharmaceutical Ga-PSMA-11 for detecting neoplastic changes in the prostate, compared to MRI and PET, without the use of the radiopharmaceutical Ga-PSMA-11 [31]. MRI sensitivity was significantly lower than the PSMA-PET (60% vs. 90%). However, the two PET methods examined were more similar (76% vs. 90%), and no statistically significant differences were observed in the sample available to the researchers.

The possibility of using radiological markers of the PSMA antigen in diagnosing prostate cancer in combination with PET scanning (21.) or with multiparametric magnetic resonance imaging mpMRI and PET scanning (22.) is also being analyzed in two ongoing studies [32,33].

The combination of PET and MRI scanning and the use of the radiopharmaceutical Ga-PSMA-11 also allows for the treatment of single lesions that appear as a result of recurrence, using ultrasound (focused ultrasound) without surgical intervention (23.) [34].

In Sweden, the Stockholm3 diagnostic protocol was developed, which is based on the examination of plasma protein markers, genetic tests and a clinical interview, and is characterized by greater specificity than PSA testing and allows unnecessary biopsies to be avoided (24.) [35].

A longitudinal, five-year study is also underway on patients with diagnosed hormone-dependent prostate cancer (25.) treated with hormone therapy (androgen deprivation—ADT). The progression of cancer is analyzed depending on the treatment regimen used [36].

In relation to stage III or IV cancer, studies are being conducted on the correlation of genetic test results with the response of patients to the pharmacological supportive treatment used (26.). Both the correlations between the effectiveness of drugs and the emerging undesirable side effects are being analyzed [37].

## 4. Discussion

Prostate cancer diagnostics in clinical practice is based on prostate-specific antigen PSA testing, rectal examination, transrectal ultrasound of the prostate gland and magnetic resonance imaging (MRI). Procedures in which a high PSA concentration is considered an indication for biopsy involve a diagnostic procedure that is, on the one hand, burdensome for the patient and, on the other hand, increases the risk of infection. In addition, many biopsies performed in a classical manner (so-called sampling), based on such indications, give negative results. The specificity of PSA testing was found to be limited and, if combined with liberal criteria for performing a biopsy, may lead to unnecessary treatment with side-effects seriously limiting a patient’s life quality, as a consequence of impotence, urinary leakage and bowel dysfunction [38]. Therefore, it raises ethical issues [39]. The likelihood of overdiagnosing is associated with comorbidity and the patients’ age [40]. Systematic continuous PSA screening, establishing a baseline for a patient and then, if an interval change was observed, deciding on the diagnostic procedures to follow, was demonstrated to be a better practice [41]. Also, researchers are looking for new biomarkers of liquid biopsy. It was demonstrated that a combination of S100A4, MRC2 and PCA3 increased the ability to discriminate between patients and controls and between different more and less aggressive stages. PCA3 itself allowed a sensitivity of 97.47%, and the use of S100A4 allowed a specificity of 90.32% [42]. Another biomarker, PSA’s “Glycan Score”, accurately indicated the PCa status without any error in a study conducted last year [43].

In response to the current state of affairs, researchers are looking for better, more specific screening methods. Therefore, the sensitivity and specificity of MRI, three-dimensional MRI, multiparametric mpMRI [44], imaging based on the combination of PET and CT scan results and PET using the radiopharmaceutical Ga-PSMA-11 have been analyzed. The benefits associated with the use of these advanced imaging methods and the costs of their use are still the subject of analysis. In the paper addressing the cost-effectiveness of magnetic resonance imaging in prostate cancer screening [45], magnetic resonance imaging (MRI) with combinations of targeted biopsy (TBx) and systematic biopsy (SBx) for early prostate cancer detection in Sweden was demonstrated to be the optimal choice on the basis of a microsimulation model as it reduced the number of biopsies across a lifetime and allowed for better cost-effectiveness of the diagnostic procedures involved. Also, it was demonstrated, on a hypothetical cohort of 448 English men, that conducting an MRI before deciding on performing a biopsy can be beneficial in terms of fewer deaths, fewer biopsies and fewer overdiagnosed cancers [46]. The experience of overdiagnosis and overtreatment may have a profound effect on psychological well-being, families, employment and life choices in general but also may lead to distrust towards conventional medicine [47].

The use of modern imaging methods gives hope for progress in the diagnostics of not only new cases of prostate cancer but also for improving the effectiveness of treatment of cases already diagnosed. On the other hand, the possibility of using the determination of the genetic profile characteristic of prostate cancer or the testing of plasma protein markers in diagnostics is also being analyzed. As more than 140 genetic variants are associated with a higher risk of prostate cancer [48], polygenic risk scores need to be used to evaluate genetic disposition to the disease. The use of polygenic risk scores also has the potential to identify those with a lower lifetime risk of developing prostate cancer than the average man, which can help reduce the number of biopsies as well. However, ethical issues need to be addressed, specifically familial implications of genetic information and the protection of genetic privacy [49].

Due to the high burden on patients taking part in diagnostic tests, on one hand, research is being undertaken to improve the effectiveness of diagnostic procedures and, on the other, methods are being sought to increase patient participation in tests. In this area, the effectiveness of educational procedures and involving patients in active participation in decision-making regarding diagnostic procedures is being analyzed. The effectiveness of delivering crucial information to patients for involving them in an informed decision-making process has been demonstrated [50]. Also, community events promoting prostate cancer screening were assessed and demonstrated to be effective. The majority of men participating engaged in medical activities like making a doctor’s appointment after participating in community events [51].

The area of interest of researchers primarily includes higher-risk populations, such as African American men and populations of people with limited access to medical services, such as people living in rural areas. For African American men, a specific scale was developed for measuring health-related decision self-efficacy regarding prostate cancer screening as a tool for developing more effective health behavior and decision-making interventions [52].

In Poland, knowledge about risk factors, diagnostic procedures and symptoms is provided to patients, for example, as part of the “I plan a long life” campaign conducted by the Ministry of Health as part of the National Oncology Strategy for 2020–2030, covering health education, health promotion and prevention.

## 5. Conclusions

Research and screening primarily focus on high-risk groups, often associated with existing social inequalities in health, such as geographically excluded people. The most widely used diagnostic methods, such as per rectal examination and transrectal ultrasound, are associated with reluctance on the part of men, which may result in them refraining from screening. For this reason, research is being carried out on the possibility of using other methods, such as three-dimensional MRI and multi-parametric mpMRI, which are less invasive. Additionally, more and more researchers are focusing on the possibility of using genetic profiling. When participating in screening tests, the challenge, as with other cancers, is to increase reporting among those at risk.

Early detection of prostate cancer significantly increases the possibility of complete recovery. For this reason, it is important to increase activities aimed at covering the at-risk population with screening as widely as possible. The factor that should be taken into account when developing new early detection methods is primarily patient comfort and the minimally invasive nature of procedures.

The current paper is based on trials registered in the ClinicalTrials.Gov registry, which is the largest database containing information on clinical trials available [53]. However, since not all studies are registered in this database, our approach has the limitation of not including unregistered studies [54]. The register is U.S.-based; however, the majority of the studies registered (56%) are non-U.S.-based regarding the location [55], which reduces the potential geographical base. Currently, the register lists studies located in 229 countries and territories.

## Figures and Tables

**Table 1:** Distribution of clinical trial analysis results in the ClinicalTrial.Gov registry according to adopted categories.

No.	Group	Title	Years	*n*	Status	Study	Population
1.	Behavioral	Smart Prostate Specific Antigen (PSA) Screening Study [12]	2021–2024	49	active	interventional	adult men PSA (\>4.0 ng/mL)
2.	interventions	Evaluating the Shared Decision Making Process Scale in Cancer Screening Decisions [13]	2020–2021	240	completed	observational	men aged 45–74
3.		Trial of Community Health Worker-led Decision Coaching [14]	2019–2023	162	completed	interventional	adult men of African American origin
4.		Fostering Shared Decision-making About Prostate Cancer Screening Among Clinicians and African American Men [15]	2020–2023	161	completed	interventional	adult men of African American origin aged at least 40
5.		Randomized Trial of Trust in Online Videos About Prostate Cancer [16]	2021–2022	3649	completed	interventional	adults aged at least 40
6.		Prostate Cancer Genius App Education and Home-based PSA Screening for African American Men [17]	2023–2025	80	active	interventional	adult men of African American origin aged 55–69
7.		The Peer Genetic Study [18]	2020–2024	149	active	interventional	adult men of African American origin aged 35–69
8.		Clinical Outcomes for Offering Genetic Testing in a Tiered Approach [19]	2020–2021	6	completed	interventional	adults aged 18–65
9.		KanSurvive: Testing a Model for Improving Cancer Survivorship Care in Rural Practice [20]	2020–2023	267	completed	interventional	adults aged 18–75
10.		Family History App in Personalized Medicine [21]	2021–2024	627	active	interventional	adults aged 30–69
11.		Adapting Multiple Behavior Interventions That Effectively Improve Cancer Survivor Health [22]	2020–2024	603	active	interventional	people aged at least 50
12.	Diagnostic	Evaluation of an MRI-based Prostate Cancer Screening Program [23]	2019–2023	241	completed	interventional	adult men
13.	tests	ReIMAGINE Prostate Cancer Screening [24]	2019–2021	309	completed	interventional	adult men
14.		Prostate Cancer Detection Screening MRI Protocol [25]	2019–2020	48	completed	interventional	men aged 18–80
15.		The BARCODE 1 Study (Full Study): The Use of Genetic Profiling to Guide Prostate Cancer Targeted Screening [26]	2019–2028	4700	active	observational	men aged 55–69
16.		Novel Synthetic T2W MR Imaging and Spin Parameter Mapping Techniques for Screening Prostate Cancer [27]	2021–2025	33	active	interventional	men aged at least 18
17.		68Ga-PSMA-11 PET/CT Screening Prior to 177Lu-PSMA-617 Therapy for Patients With Metastatic Castrate Resistant Prostate Cancer [28]	2022–2023	163	completed	interventional	men aged at least 18
18.		PReclude Infection EVEnts With No Prophylaxis Transperineal Biopsy [29]	2021–2024	738	completed	interventional	men aged at least 18
19.		Telomere Associated Variables (TAVs) in Prostate Cancer [30]	2019–2023	509	completed	observational	adult men referred for a biopsy based on PSA test results
20.		PSMA-PET for Biopsy and Treatment Guidance in Primary Prostate Cancer [31]	2019–2022	36	completed	interventional	men aged at least 18
21.		[Al18F] PSMA137 PET/CT Imaging for PSMA-Positive Cancer Patients [32]	2021–2024	20	active	interventional	men aged at least 18
22.		PSMA-PET/MRI Unfavorable-Risk Target Volume Pilot Study [33]	2020–2028	9	active	interventional	men aged at least 18
23.		Feasibility Study on the Use of PET-MRI/68Ga-PSMA Imaging for HIFU-focal Treatment in the Event of Recurrent Prostate Cancer After Radiotherapy—a PSMA Study [34]	2020–2020	11	completed	interventional	men aged at least 50
24.		Stockholm3 Validation Study in a Multi-Ethnic Cohort [35]	2019–2023	2152	completed	observational	men aged 45–75
25.		A Registry Study to Observe Clinical Outcomes of Participants With High-risk Metastatic Hormone-naïve Prostate Cancer in Japan [36]	2019–2025	979	active	observational	men aged at least 20 with diagnosed hormone-dependent prostate cancer
26.		Pharmacogenomics Testing in the Optimal Use of Supportive Care Medications in Stage III-IV Cancer [37]	2019–2022	197	completed	interventional	patients in stage III or IV of cancer

## Data Availability

Data available at authors.

## References

[1] WHO International Agency for Research on Cancer. https://gco.iarc.who.int/media/globocan/factsheets/cancers/27-prostate-fact-sheet.pdf.

[2] European Commission Prostate Cancer Burden in EU-27. https://ecis.jrc.ec.europa.eu/sites/default/files/2023-12/prostate_cancer_En-Nov_2021.pdf.

[3] American Cancer Society Prostate Cancer Risk Factors. https://www.cancer.org/cancer/types/prostate-cancer/causes-risks-prevention/risk-factors.html.

[4] Al-Fayez S., El-Metwally A. (2023). Cigarette smoking and prostate cancer: A systematic review and meta-analysis of prospective cohort studies. Tob. Induc. Dis..

[5] Hong S., Khil H., Lee D.H., Keum N., Giovannucci E.L. (2020). Alcohol Consumption and the Risk of Prostate Cancer: A Dose-Response Meta-Analysis. Nutrients.

[6] US Preventive Services Task Force Prostate Cancer: Screening. https://www.uspreventiveservicestaskforce.org/uspstf/recommendation/prostate-cancer-screening.

[7] https://uroweb.org/guidelines/prostate-cancer/chapter/diagnostic-evaluation.

[8] https://www.annalsofoncology.org/article/S0923-7534(20)39898-7/fulltext.

[9] https://www.hopkinsmedicine.org/health/conditions-and-diseases/prostate-cancer/prostate-cancer-prevention.

[10] https://clinicaltrials.gov/.

[11] Fudali K.., Sagan K., Kwiatkowska E., Kosendiak A. (2023). The impact of the early period of the COVID-19 pandemic on screening programmes of breast, colorectal and cervical cancer. J. Health Inequalities.

[12] https://clinicaltrials.gov/study/NCT04782713?titles=Smart%20Prostate%20Specific%20Antigen%20(PSA)%20Screening%20Study%20&rank=1.

[13] https://clinicaltrials.gov/study/NCT04601272?titles=Evaluating%20the%20Shared%20Decision%20Making%20Process%20Scale%20in%20Cancer%20Screening%20Decisions%20&rank=1.

[14] https://clinicaltrials.gov/study/NCT03726320?titles=Trial%20of%20Community%20Health%20Worker-led%20Decision%20Coaching%20&rank=1.

[15] https://clinicaltrials.gov/study/NCT03869216?titles=Fostering%20Shared%20Decision-making%20About%20Prostate%20Cancer%20Screening%20&rank=1.

[16] https://clinicaltrials.gov/study/NCT05886751?titles=Randomized%20Trial%20of%20Trust%20in%20Online%20Videos%20About%20Prostate%20Cancer%20&rank=1.

[17] https://clinicaltrials.gov/study/NCT05331638?titles=Prostate%20Cancer%20Genius%20App%20Education%20and%20Home-based%20PSA%20Screening%20for%20African%20American%20Men%20&rank=1.

[18] https://clinicaltrials.gov/study/NCT05011799?titles=The%20Peer%20Genetic%20Study%20&rank=1.

[19] https://clinicaltrials.gov/study/NCT04902144?titles=Clinical%20Outcomes%20for%20Offering%20Genetic%20Testing%20in%20a%20Tiered%20Approach%20&rank=1.

[20] https://clinicaltrials.gov/study/NCT04763824?titles=KanSurvive:%20Testing%20a%20&rank=1.

[21] https://clinicaltrials.gov/study/NCT04726319?titles=Family%20History%20App%20in%20Personalized%20Medicine%20&rank=1.

[22] https://clinicaltrials.gov/study/NCT04000880?titles=Adapting%20Multiple%20Behavior%20Interventions%20&rank=1.

[23] https://clinicaltrials.gov/study/NCT03749993?titles=Evaluation%20of%20a%20MRI-based%20Prostate%20Cancer%20&rank=1.

[24] https://clinicaltrials.gov/study/NCT04063566?titles=ReIMAGINE%20Prostate%20Cancer%20Screening%20&rank=1.

[25] https://clinicaltrials.gov/study/NCT04175730?titles=Prostate%20Cancer%20Detection%20Screening%20MRI%20Protocol%20&rank=1.

[26] https://clinicaltrials.gov/study/NCT03857477?titles=The%20BARCODE%201%20Study%20&rank=2.

[27] https://clinicaltrials.gov/study/NCT05055843?titles=Novel%20Synthetic%20T2W%20MR%20Imaging%20and%20&rank=1.

[28] https://clinicaltrials.gov/study/NCT05547386?titles=68Ga-PSMA-11%20PET%2FCT%20Screening%20Prior%20to%20177Lu-PSMA-617%20&rank=1.

[29] https://clinicaltrials.gov/study/NCT04843566?titles=PReclude%20Infection%20EVEnts%20With%20No%20Prophylaxis%20Transperineal%20Biopsy%20&rank=1.

[30] https://clinicaltrials.gov/study/NCT04124900?titles=Telomere%20Associated%20Variables&rank=1.

[31] https://clinicaltrials.gov/study/NCT03429244?titles=PSMA-PET%20for%20Biopsy%20and%20Treatment%20Guidance%20in%20Primary%20Prostate%20Cancer%20&rank=1.

[32] https://ctv.veeva.com/study/al18f-psma137-pet-ct-imaging-for-psma-positive-cancer-patients.

[33] https://clinicaltrials.gov/study/NCT04176497?titles=PSMA-PET%2FMRI%20Unfavorable-Risk%20Target%20Volume%20Pilot%20Study%20&rank=1.

[34] https://clinicaltrials.gov/study/NCT03927521?titles=Feasibility%20Study%20on%20the%20Use%20of%20PET&rank=2.

[35] https://clinicaltrials.gov/study/NCT04583072?titles=Stockholm3%20Validation%20Study%20in%20a%20Multi-Ethnic%20&rank=1.

[36] https://clinicaltrials.gov/study/NCT04034095?titles=Hormone-na%C3%AFve%20Prostate%20Cancer%20in%20Japan%20&rank=1.

[37] https://clinicaltrials.gov/study/NCT04067960?titles=Pharmacogenomics%20Testing%20in%20the%20Optimal%20Use%20of%20Supportive%20Care%20Medications%20&rank=1.

[38] Carlsson S.V., Lilja H. (2019). Perspective on Prostate Cancer Screening. Clin. Chem..

[39] Mishra S.C. (2021). A discussion on controversies and ethical dilemmas in prostate cancer screening. J. Med. Ethics.

[40] Gulati R., Psutka S.P., Etzioni R. (2019). Personalized Risks of Over Diagnosis for Screen Detected Prostate Cancer Incorporating Patient Comorbidities: Estimation and Communication. J. Urol..

[41] Vilson F.L., Li S., Brooks J.D., Eisenberg M.L. (2020). Sudden PSA rise to ≥20 ng/ml and prostate cancer diagnosis in the United States: A population-based study. Prostate.

[42] Alvarez-Cubero M.J., Arance E., de Santiago E., Sanchez P., Sepúlveda M.R., Marrero R., Lorente J.A., Gonzalez-Cabezuelo J.M., Cuenca-Lopez S., Cozar J.M. (2023). Follow-Up Biomarkers in the Evolution of Prostate Cancer, Levels of S100A4 as a Detector in Plasma. Int. J. Mol. Sci..

[43] Díaz-Fernández A., Ryø Jochumsen M., Christensen N.L., Dalsgaard Sørensen K., Bouchelouche K., Borre M., Holm Vendelbo M., Ferapontova E.E. (2024). Liquid-Biopsy Glycan Score Biomarker Accurately Indicates and Stratifies Primary and Metastatic Prostate Cancers. Anal. Chem..

[44] Launer B.M., Ellis T.A., Scarpato K.R. (2025). A contemporary review: mpMRI in prostate cancer screening and diagnosis. Urol. Oncol..

[45] Hao S., Karlsson A., Heintz E., Elfström K.M., Nordström T., Clements M. (2021). Cost-Effectiveness of Magnetic Resonance Imaging in Prostate Cancer Screening: A Microsimulation Study. Value Health.

[46] Callender T., Emberton M., Morris S., Pharoah P.D.P., Pashayan N. (2021). Benefit, Harm, and Cost-effectiveness Associated with Magnetic Resonance Imaging Before Biopsy in Age-based and Risk-stratified Screening for Prostate Cancer. JAMA Netw. Open.

[47] McCaffery K., Nickel B., Pickles K., Moynihan R., Kramer B., Barratt A., Hersch J. (2019). Resisting recommended treatment for prostate cancer: A qualitative analysis of the lived experience of possible overdiagnosis. BMJ Open.

[48] Byrne L., Toland A.E. (2021). Polygenic Risk Scores in Prostate Cancer Risk Assessment and Screening. Urol. Clin. N. Am..

[49] Emanuel E.J., Joffe S., Kufe D.W., Pollock R.E., Weichselbaum R.R., Bast R.C., Gansler T.S., Holland J.F., Frei E. (2003). Ethical Issues in Cancer Genetics. Holland-Frei Cancer Medicine.

[50] Dierks T., Heijnsdijk E.A.M., Korfage I.J., Roobol M.J., de Koning H.J. (2019). Informed decision-making based on a leaflet in the context of prostate cancer screening. Patient Educ. Couns.

[51] Drake B.F., Lewis-Thames M.W., Brown A., Rancilio D., Hicks V. (2019). An Evaluation of Follow-Up Activities of Participants from an Urban Prostate Cancer Screening Event. Am. J. Men′s Health.

[52] Owens O.L., Wooten N.R., Tavakoli A.S. (2020). Adaptation and Initial Psychometric Evaluation of an Informed Prostate Cancer Screening Decision Self-Efficacy Scale for African-American Men. J. Racial Ethn. Health Disparities.

[53] Fine A. Marking a Milestone: The Modernized ClinicalTrials.gov Becomes the Singular Website Experience 2023. https://nexus.od.nih.gov/all/2024/06/06/marking-a-milestone-the-modernized-clinicaltrials-gov-becomes-the-singular-website-experience/.

[54] Tse T., Fain K.M., Zarin D.A. (2018). How to avoid common problems when using ClinicalTrials.gov in research: 10 issues to consider. BMJ.

[55] Trends and Charts on Registered Studies. https://clinicaltrials.gov/about-site/trends-charts.

